# Editorial: Low-Intensity Interventions for Psychiatric Disorders

**DOI:** 10.3389/fpsyt.2020.619871

**Published:** 2020-11-25

**Authors:** Marit Sijbrandij, Annet Kleiboer, Saeed Farooq

**Affiliations:** ^1^Department of Clinical, Neuro- and Developmental Psychology, Amsterdam Public Health Institute, Vrije Universiteit, Amsterdam, Netherlands; ^2^World Health Organization Collaborating Center for Research and Dissemination of Psychological Interventions, Vrije Universiteit, Amsterdam, Netherlands; ^3^School of Medicine, Keele University and Midlands Partnership NHS Foundation Trust, Staffordshire, United Kingdom

**Keywords:** depression, anxiety, task-shifting and task-sharing, low-intensity treatments, global mental health, psychological interventions

Essentially, low-intensity interventions are treatments with a low usage of “specialist therapist” time (1). These include a broad variety of interventions such as group interventions, brief versions of individual therapies (i.e., not more than approximately six sessions), interventions supported by self-help materials or internet-based platforms (1, 2), or task-shifting interventions that are delivered by non-specialists such as paraprofessionals or lay health workers. The advantages of low intensity interventions are obvious: shorter and less time-intensive treatments are cheaper and easier to integrate into primary and community health care settings, or within stepped care models. This may increase the access to mental health interventions for larger numbers of people in need of mental health care. However, low-intensity treatments also have challenges and disadvantages. For example, relapse rates with low-intensity treatment for anxiety and depression are relatively high (3), it is yet unknown for which patients low-intensity treatments are most suited, and -integrated into a stepped-care system- unsuccessful low-intensity treatment may discourage patients in pursuing a high-intensity treatment.

During the past decade, the evidence for low-intensity interventions for psychiatric disorders has been accruing. Global mental health researchers have put the worldwide mental health treatment gap on the scientific agenda, referring to the enormous difference between people in need and the people actually receiving mental health care (4). This gap is highest in low-income settings where resources for providing mental health care are scarce. Alongside to educating higher numbers of mental health professionals, and increasing budget for mental health expenditures, task-shifting is another strategy to increase access to mental health care. Recent randomized controlled trials (RCTs) in low-resource settings such as Pakistan (5) and Kenya (6) have shown positive effects of low-intensity task-shifting strategies in reducing symptoms of depression, anxiety, and posttraumatic stress disorder. Less is known about whether scaling up low-intensity interventions improves access to mental health care, but studies are underway [see (7)].

Building on this largely positive evidence base, scalable task-shifting interventions are currently developed for other target populations. The Research Topic papers of Brown et al., Atif et al., and Machuka et al. provide examples of how the work on task-shifting strategies in low-resource settings is evolving. Early Adolescent Skills for Emotions (EASE) is a World Health Organization (WHO) transdiagnostic program based on principles of Cognitive Behavioral Therapy (CBT) and Problem Solving Therapy (PST), addresses psychological distress in young adolescents and is delivered by lay therapists (Brown et al.). Brown et al. provide a useful template on how to conduct a rigorous cultural adaptation of low intensity interventions. Similar work was done in Pakistan, where Atif et al. developed “Happy Mother, Healthy Baby,” to address anxiety during pregnancy, an important precursor of postnatal depression. Their paper describes the qualitative work guiding the development of the intervention and the adaptation to the Pakistani context. They found that it was acceptable, feasible, and perceived to be helpful by the women receiving it (Atif et al.). Finally, a somatic condition associated with considerable distress and impairment, is the diagnosis of Human Immunodeficiency Virus/Acquired Immune Deficiency Syndrome (HIV/AIDS). In Kenya, Machuka et al. developed an intervention to improve well-being and treatment adherence in children and young people with a (HIV/AIDS) diagnosis. Their low-intensity intervention consists of eight support-group sessions led by a facilitator (Machuka et al.).

A specific set of low intensity interventions are the digital and e-mental health interventions. Most of the evaluations of e-mental health programs has been done in high-income settings, but exciting new studies in low-income settings are now appearing along with the increased use of digital media. WHO's Step-by-Step, described by Harper Shehadeh et al., is available in Arabic and English through smartphone, tablet, or computer, and is based on CBT and PST strategies. An uncontrolled pilot evaluation in Lebanon found that over time, symptoms of depression improved in participants, and the content of the intervention itself and the digital delivery mode to be acceptable and feasible. However, there was also significant attrition, predominantly due to lack of motivation and technical problems. This is a common complicating factor when implementing e-mental health interventions. To see which participants drop-out from low-intensity e-health intervention, Kazlauskas et al. investigated predictors to adherence to a brief (1 to 3 h) internet self-help intervention for adjustment disorder in Lithuania, a high-income country but with limited access to mental health interventions. The intervention included body relaxation, mindfulness, time management, and resolving conflicts in interpersonal relationships. The authors found a number of factors to be important in predicting adherence, such as being female, being older, having high expectations and already following regular face-to-face treatment.

In addition to task-shifting and digital advances, innovations in content and delivery of low-intensity interventions also found a place in this Research Topic. For example, Kong et al. provided an overview of the use of Tai Chi in regulating emotion, and its potential to be used as a low intensity intervention for depression. Accessible virtual reality (VR) technologies, such as the use of consumer VR glasses, provide interesting new opportunities to scale up VR interventions to larger groups of patients. The first generation of VR interventions were high-intensity treatments for anxiety disorders, phobias and PTSD often requiring large installations located in research or treatment centers (8). Although still in its early stages, Lindner, Hamilton et al. describe how VR could be used to deliver a low-intensity intervention for depression. In a second paper in this Research Topic, Lindner, Miloff et al. describe their uncontrolled pilot study on the effects of a single-session automated low-intensity VR intervention with gaming elements to reduce spider phobia in psychology students. The intervention was well-tolerated, and results were promising. This will, however, require replication in a larger RCT (Lindner, Miloff et al.).

In sum, low-intensity interventions are to stay, both in the clinic and in the community. With the current COVID-19 pandemic challenging the mental health systems of countries worldwide, the availability of highly scalable strategies to deliver interventions remotely and/ or digitally to people in need of psychological treatments is as timely as ever.

## Author Contributions

All authors listed have made a substantial, direct and intellectual contribution to the work, and approved it for publication.

## Conflict of Interest

The authors declare that the research was conducted in the absence of any commercial or financial relationships that could be construed as a potential conflict of interest.
